# ATP Modifies the Proteome of Extracellular Vesicles Released by Microglia and Influences Their Action on Astrocytes

**DOI:** 10.3389/fphar.2017.00910

**Published:** 2017-12-13

**Authors:** Francesco Drago, Marta Lombardi, Ilaria Prada, Martina Gabrielli, Pooja Joshi, Dan Cojoc, Julien Franck, Isabelle Fournier, Jacopo Vizioli, Claudia Verderio

**Affiliations:** ^1^Univ. Lille, INSERM, U1192 – Protéomique Réponse Inflammatoire Spectrométrie de Masse – PRISM, Lille, France; ^2^Fondazione Istituto Oncologico del Mediterraneo, Viagrande, Italy; ^3^IRCCS Humanitas, Rozzano, Italy; ^4^Institute of Neuroscience (CNR), Milan, Italy; ^5^Institute of Materials (CNR), Trieste, Italy

**Keywords:** ATP, microglia, extracellular vesicles, proteomics, astrocyte activation

## Abstract

Extracellular ATP is among molecules promoting microglia activation and inducing the release of extracellular vesicles (EVs), which are potent mediators of intercellular communication between microglia and the microenvironment. We previously showed that EVs produced under ATP stimulation (ATP-EVs) propagate a robust inflammatory reaction among astrocytes and microglia *in vitro* and in mice with subclinical neuroinflammation (Verderio et al., 2012). However, the proteome of EVs released upon ATP stimulation has not yet been elucidated. In this study we applied a label free proteomic approach to characterize the proteome of EVs released constitutively and during microglia activation with ATP. We show that ATP drives sorting in EVs of a set of proteins implicated in cell adhesion/extracellular matrix organization, autophagy-lysosomal pathway and cellular metabolism, that may influence the response of recipient astrocytes to EVs. These data provide new clues to molecular mechanisms involved in microglia response to ATP and in microglia signaling to the environment via EVs.

## Introduction

Microglia are essential components of the innate immune response in the brain. They are self-renewing, long-lived cells, and stem from a unique non-haematopoietic yolk-sac-derived cell lineage (Ginhoux et al., 2010). They are multitasking cells involved in various functions under physiological and pathological states, participating in synaptic refinement, phagocytosis or immunosurveillance (Casano and Peri, 2015). During brain development microglia regulate the formation and stability of dendritic spines and eliminate via phagocytosis redundant synapses, a process known as synaptic pruning (Paolicelli et al., 2011). This process involves the complement factors C1q and C3, which localize to redundant synapse, and C3 receptor, which triggers synaptic engulfment (Stevens et al., 2007). In the adult brain, microglia are critical for the maintenance of brain homeostasis and continuously move their processes to survey the surrounding territory (Davalos et al., 2005) (Nimmerjahn et al., 2005). In response to injury or infection, these highly dynamic cells proliferate and migrate to sites of injury, where they participate in mechanisms of injury but also in tissue repair.

Extracellular ATP is among molecules promoting microglia activation, proliferation, phagocytic function and guiding their migration toward damaged cells (Dou et al., 2012; Sieger et al., 2012; Domercq et al., 2013). ATP accumulates extracellularly at sites of injury or inflammation, being released from dead cells (Di Virgilio, 2007). By inducing further release of ATP from neighboring cells, the molecule establishes a long-range ATP gradient, that induces chemotaxis of remote microglia (Honda et al., 2001; Corriden and Insel, 2012; Casano et al., 2016).

Our previous evidence indicates that ATP, through activation of the ATP receptor P2X7, massively increases release of EVs from microglia (Bianco et al., 2005b, 2009). EVs are membrane vesicles released by all cells which contain a selection of donor cell components, including proteins, lipids and RNA, and serve as transfer vehicles for these molecules between cells. By exposing cell-type-specific adhesion receptors, EVs interact with specific cells and deliver complex “signals,” playing a key role in cell-to-cell signaling. EVs have different sizes and subcellular origin. Quite large EVs bud from the plasma membrane (ectosomes, also called microvesicles or MVs) while small EVs result from exocytosis of multivesicular bodies (exosomes) (Cocucci and Meldolesi, 2015).

Extracellular vesicles released by ATP-stimulated microglia induce a robust inflammatory reaction in glial cells *in vitro* and propagate an inflammatory response among microglia in mice with subclinical neuroinflammation (Verderio et al., 2012). However, the action of ATP-EVs has never been compared to that of constitutive EVs nor the proteome of constitutive or ATP-EVs has been elucidated yet (Prada et al., 2013). To our knowledge, only one proteomic study has been performed on EVs derived from primary microglia. This work led to the identification of ∼45 proteins in exosomes released from microglia activated with the signaling protein Wnt3a but it did not identify any protein in constitutive exosomes (Hooper et al., 2012), thus limiting current knowledge of EV composition.

In this study we applied a label free proteomic approach to explore the changes in EV proteome induced by microglia activation with ATP. We also investigated how ATP stimulation impacts the response of recipient astrocytes to microglia-derived EVs. We found that ATP stimulation drives secretion via EVs of a set of proteins implicated in cell adhesion/extracellular matrix organization, in degradative pathways, and energy metabolism, and that ATP-EVs enhance the expression of few activation markers in target astrocytes. These data provide new clues to molecular mechanisms involved in microglia response to ATP and in their signaling to the environment.

## Materials and Methods

### Animals

All the experimental procedures followed the guidelines established by the European Legislation (Directive 2010/63/EU) and the Italian Legislation (L.D. no 26/2014).

### Primary Glial Culture and Stimulation

Mixed glial cell cultures, containing both astrocytes and microglial cells, were established from postnatal rat Sprague–Dawley pups (P2). Briefly, after dissection, hippocampi and cortices were dissociated by treatment with trypsin and DNase-I for 15 min at 37°C, followed by fragmentation with a fire-polished Pasteur pipette. Dissociated cells were plated on poly-L-lysine coated T75 flasks in minimal essential medium (E-MEM, Invitrogen) supplemented with 20% fetal bovine serum (Gibco, Life Technologies, Carlsbad, CA, United States) and glucose (5.5 g/L). To obtain a pure astrocyte monolayer, microglial cells were harvested from 7-days-old cultures by orbital shaking for 30 min at 1300 rpm. Astrocytes were trypsinised and re-plated onto poly-L-lysine-coated glass coverslips while shaken microglia were re-plated on poly-DL-ornithine-coated tissue culture dishes.

Recipient astrocytes were exposed to an amount of EVs produced by twice as many donor microglia (1:2 receiving cells to donor cells relative ratio). To reduce the level of activation, recipient astrocytes were pre-starved overnight in serum-free medium and kept in low (1%) serum medium during exposure to EVs. To minimize the activation of microglia, half of the medium in which microglia were kept after shaking from mixed glial cultures was replaced with fresh low (1%) serum medium. At the end of incubation, recipient astrocytes were washed and harvested with TRIZOL for RT-PCR analysis.

### EV Isolation and Quantification

Extracellular vesicles released from 1 × 10^6^ microglia constitutively or upon exposure to 1 mM ATP for 1h in KRH (125 mM NaCl, 5 mM KCl, 1.2 mM MgSO_4_, 1.2 mM KH_2_PO_4_, 2 mM CaCl_2_, 6 mM D-glucose, and 25 mM HEPES/NaOH, pH 7.4) were pelletted at 10K g (ectosome-enriched fraction) and 100K g (exosomes-enriched fraction) after pre-clearing from cells and debris as described previously (Gabrielli et al., 2015). TRPS, by qNano (Izon, Christchurch, New Zealand) was used to measure the size distribution and concentration of particles in 10 and 100K g pellets after re-suspension in 100 μl. TRPS is an impedance based method. A voltage is applied across a pore that is filled with electrolyte, resulting in an ionic current. As EVs cross the pore they briefly block the ionic current, creating a blockade event, which is proportional to EV volume. A reagent kit from Izon (Izon EV reagent kit) were used for both pre-treating the pore and suspending EVs in order to prevent EV binding to the pore or spontaneous EV aggregation. NP300 nanopore (150–600 nm diameter range; Izon) was used for MV sample analysis, while NP150 nanopore (85–300 nm diameter range; Izon) was used for exosome sample analysis. In each experiment, the same applied voltage, pressure and pore stretch values were set for all MV/exosome sample recordings and relative calibration. Three pressure values per sample were used for multipressure analysis. CPC200 and CPC150 calibration particles (carboxylated polystyrene particles, supplied by Izon and diluted following manufacturer’s instructions) were used as standards, for MV and exosome sample respectively. They were measured immediately before or after the experimental samples under identical conditions. Data acquisition and analysis were performed using Izon Control Suite software (version V3.2).

To deplete luminal cargo, EVs were broken by freeze and thaw and repelletted at 100K g for 1 h. To mask PS residues on the EV surface and avoid contact with recipient astrocytes, EVs were resuspended in annexin-V for 30 min, washed and repelleted. For biochemical fractionation of EVs, total lipids were extracted through the method previously described (Antonucci et al., 2012) with 2:1 (by volume) of chloroform and methanol. The lipid fraction was evaporated under a nitrogen stream, dried for 1 h at 50°C and resuspended in PBS at 40°C in order to obtain multilamellar vesicles. Small unilamellar vesicles were obtained by sonication, following the procedure of (Barenholz et al., 1977).

### EV Proteomics

Extracellular vesicles released from 15 × 10^6^ microglia constitutively or upon exposure to 1 mM ATP in MEM for 1h were centrifuged as above and frozen at -80°C. Dried samples were reconstituted with 20 μL of 50 mM bicarbonate buffer containing 50 mM DTT and 4% SDS. The samples were then loaded on 12% polyacrylamide gel and separated at 70 V for 15 min and then 120 V until the dye front entered in the separating gel at a distance of 1 cm. The gel was cut into pieces of 1 mm^3^. Pieces were washed with 300 μl of distilled deionized water for 15 min, 300 μl of acetonitrile (ACN) for 15 min, and 300 μl of NH_4_HCO_3_ 100 mM (pH 8) for 15 min. Then a mix of 300 μl of NH_4_HCO_3_/ACN (1:1, v/v) for 15 min and 300 μl of ACN for 5 min. Band pieces were dried in a Speedvac for 5 min. The reduction of cysteine residues was made with 50 μl of 10 mM of DTT in NH_4_HCO_3_ 100 mM (pH 8). Pieces were incubated at 56°C for 1 h. Alkylation of cysteines was made with 50 μl of 50 mM of IAA in NH_4_HCO_3_ 100 mM (pH 8). Pieces were incubated at room temperature in the dark for 30 min. Band pieces were washed a second time with 300 μl of NH_4_HCO_3_ 100 mM (pH 8) for 15 min. Then a mix of 300 μl of NH_4_HCO_3_/ACN (1:1, v/v) for 15 min and 300 μl of ACN for 5 min. Band pieces were dried in a Speedvac for 5 min. A digestion of band pieces was made with trypsin (12.5 μg/ml) in NH_4_HCO_3_ 20 mM (pH 8), enough to cover pieces. Pieces were incubated at 37°C overnight. Peptides were extracted on shaking platform with 50 μl of FA 1% two times for 20 min, then 150 μl of ACN for 10 min. The supernatant was transferred in new tube and dried with Speedvac.

The trypsin-digested protein extracts were reconstituted with 20 μl of 5% ACN/0.1% FA and injected on an EASY-nLC 1000 UPLC (Thermo Fisher Scientific) equipped with a 75 μm × 2 cm Acclaim PepMap 100 pre-column with nanoViper fittings and a 50 μm ID × 150 mm Acclaim PepMap RSLC analytical column (C18, particle size 2 μm, pore size 100 Å, Thermo Fisher Scientific). The peptides were eluted using a 2 h gradient of ACN starting from 5 to 30% over 120 min at a flow rate of 300 nl/min. The Q-Exactive instrument was set to acquire top 10 MS2. The survey scans were taken at 70,000 FWHM (at m/z 400) resolving power in positive mode and using a target of 3E6 and default charge state of +2. Unassigned and +1 charge states were rejected and dynamic exclusion was enabled for 20 s. The scan range was set to 300–1600 m/z. For the MS2, 1 microscan was obtained at 17,500 FWHM, isolation window of 4.0 m/z and a normalized collision energy (NCE) = 30 using a scan range between 200 and 2000 m/z.

### Data Analysis

Protein tandem MS/MS data were processed using Proteome Discoverer 1.4 (Thermo Fisher Scientific). Peptides were identified by using the Sequest search engine, where target-decoy searches were performed against the *Rattus norvegicus* UniProt database (accessed March 4, 2014, 33,675 entries) combined with the 262 commonly detected contaminant databases. The parent and fragment mass tolerances were set at 10 ppm and 0.5 Da, respectively. The enzyme used was trypsin, and the maximum allowable cleavages were set to 2. Carbamidomethylation of cysteine was set as fixed modification while oxidation of methionine was set as variable modifications. FDR for the peptide and protein levels were both set at 0.01.

The data sets and Proteome Discoverer result files used for analysis were deposited at the ProteomeXchange Consortium^1^ via the PRIDE partner repository with the data set identifier PXD007650 (For reviewer access only Username: reviewer86756@ebi.ac.uk; Password: 3qSILRLn).

Proteins were clustered in categories depending on their known main biological function using two different open source bioinformatics resources: DAVID Bioinformatics Resource 6.8^2^ and PANTHER (Protein ANalysis THrough Evolutionary Relationships) database^3^. In both cases, the whole *Rattus norvegicus* genome was employed as background list. The analysis of cellular components and biological processes was performed in DAVID and selecting the GO terms for Cellular Component (GOTERM_CC_FAT) and for Biological Process (GOTERM_BP DIRECT). Molecular Function analysis was performed with AgBase Bioinformatics Resource 2.00^4^ using the AgBase GO slim viewer Molecular Function. Pathway overrepresentation analysis was performed using DAVID bioinformatics resource and comparing the representation of the different KEGG^5^ terms (KEGG_PATHWAY) to the expected pathway representation in rat. This analysis was coupled to the pathway enrichment analysis performed with PANTHER using the PANTHER Pathway keywords and exported as bar chart of representation percentages.

### EV Delivery by Optical Manipulation

An IR laser beam (1064 nm, CW) for trapping was coupled into the optical path of an inverted microscope (Axiovert200M, Zeiss) through the right port of the microscope. The trapping beam was directed to the microscope lens (Zeiss 63X, NA 1.4) by the corresponding port mirror (100%) and the tube lens. Optical trapping and manipulation of EVs was performed following the approach previously described (Prada et al., 2016). Immediately before recording, ATP-EVs or constitutive EVs (100K g pellet) were added to in the temperature controlled recording chamber, where astrocytes plated on glass coverslips, were maintained in 400 μl of medium. As soon as an EV appeared in the recording field, it was trapped and positioned on a selected astrocyte by moving the cell stage horizontally and the microscope lens axially. After about 30 s from contact, the laser was switched off to prove EV-astrocyte interaction. During the experiments astrocytes were live imaged with a spinning disk confocal microscope (UltraVIEW acquisition system, Perkin Elmer Waltham, MA, United States) using a digital camera (High Sensitivity USB 3.0 CMOS Camera 1280 × 1024 Global Shutter Monochrome Sensor, Thorlabs, Newton, NJ, United States) at a frame rate of 2 Hz.

### Reverse Transcriptase-Coupled PCR

Total RNA was isolated from rat primary astrocytes using Direct-zol^TM^ RNA MiniPrep (Zymo Research) following the manufacturer’s protocol. cDNA synthesis was performed using High Capacity cDNA Reverse Transcription Kit (Applied Biosystems) and Random Hexamers as primer. The resulting cDNAs were amplified using TaqMan^®^ Gene Expression Assay (Applied Biosystems). The mRNA expression was normalized to the label of Rpl13 (Ribosomal Protein L13) mRNA.

### Statistical Analysis

All data are presented as mean ± SE from the indicated number of independent experiments. Statistical analysis was performed using SigmaPlot 12.0 (Jandel Scientific, San Jose, CA, United States) software. After testing data for normal distribution, the appropriate statistical test has been used (see figure legends). Differences were considered significant when *P* was <0.05, *P* < 0.01 or *P* < 0.001 and they are indicated by one, two or three asterisks, respectively.

## Results

### Constitutive and ATP-Induced EV Production from Rat Primary Microglia

**FIGURE 1:**
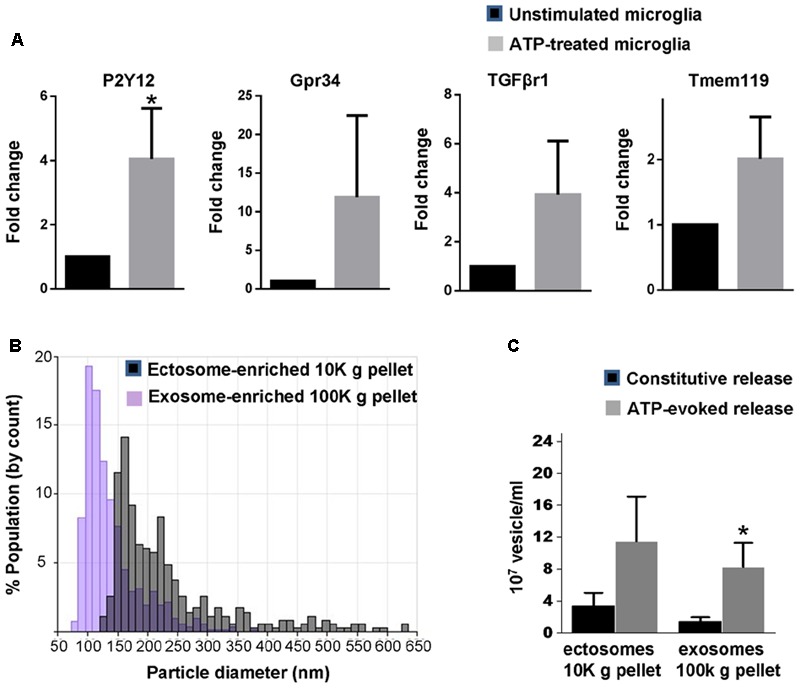
Basal and ATP-induced production of EVs in primary rat microglia. **(A)** q-PCR analysis for the adult microglia markers P2Y12, Gpr34, TGFβr1 and Tmem119 in unstimulated rat primary microglia and in cells activated with 1 mM ATP for 1h (P2Y12: Mann–Whitney Rank Sum test *P* = 0.029; Gpr34: Mann–Whitney Rank Sum test *P* = 0,343, TGFβr1: Mann–Whitney Rank Sum test *P* = 0,100; Tmem119: Mann–Whitney Rank Sum test *P* = 0.100; *N* = 3). **(B)** Representative particle size distributions of ectosome-enriched 10 Kg pellet (gray) and exosome-enriched 100 Kg pellet (violet) analyzed using TRPS. The size distributions are shown as histograms with bin width 10 nm. **(C)** Histograms show the number of EVs released constitutively or under ATP from 1 million microglia, centrifuged and re-suspended in 100 μl of 0.1 μm-filtered Krebs-Ringer solution (ATP-ectosomes versus constitutive ectosomes Mann–Whitney Rank Sum Test *P* = 0.182; *N* = 5; ATP-exosomes versus constitutive exosomes, Mann-Whitney Rank Sum Test *P* = 0.048; *N* = 3).

Microglia are equipped with several ATP receptors (Farber and Kettenmann, 2006; Pocock and Kettenmann, 2007), including P2X7 receptor, a key determinant of cellular metabolism (Adinolfi et al., 2005), which massively increases release of EV (Bianco et al., 2005b). Among ATP receptors, P2Y12 is a key marker of adult microglia (Hickman et al., 2013; Butovsky et al., 2014) and is required for homeostatic activity and phagocytosis (Ohsawa et al., 2010; Preissler et al., 2015). In order to validate new-born rat primary microglia as a suitable system to characterize EVs secreted upon ATP stimulation we checked for the presence of P2Y12 as well as other homeostatic genes enriched in adult microglia (Gpr34, TGFβr1 and Tmem119) (Butovsky et al., 2014; Buttgereit et al., 2016; Matcovitch-Natan et al., 2016) and few metabolic genes in the cultures. We found that new-born microglia constitutively express P2Y12, Gpr34, TGFβr1, Tmem119 transcripts and respond to 1 mM ATP by upregulating P2Y12 (**Figure 1A**) and few metabolic genes (Supplementary Figure 1).

We next isolated by differential centrifugation quite large ectosomes (10,000 × *g* = 10K) and smaller exosomes (100,000 × *g* = 100K) from the medium conditioned by microglia either kept under resting conditions or exposed to ATP for 1 h, as previously described (Gabrielli et al., 2015). Our previous evidence indicates that 1 h stimulation with ATP does not induce cell damage or apoptosis (Bianco et al., 2005b). Accordingly, EVs isolated under ATP stimulation are not positive for apoptotic markers (Verderio et al., 2012) nor contaminated by intracellular organelles derived from damaged cells (Gabrielli et al., 2015). Quantification by TRPS confirmed the enrichment of quite large vesicles in the 10K pellet (mean diameter = 180.00 ± 26.41 nm) and of smaller vesicles in the 100K pellet (mean diameter = 111.97 ± 12.27 nm) (**Figure 1B**). It also revealed that stimulation with ATP increases production of exosomes but not ectosomes under stimulation for 1 h (**Figure 1C**).

### Proteomic Analysis of Ectosomes and Exosomes Constitutively Released by Microglia

**FIGURE 2:**
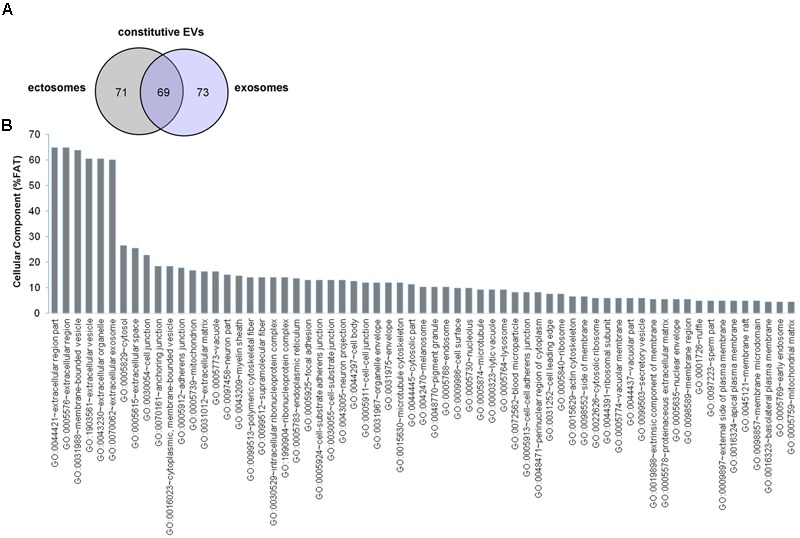
Proteome of constitutive EVs released from microglia. **(A)** Venn diagram of the numerical values for common and unique proteins present in ectosomes (gray) and exosomes (violet). **(B)** Analysis of Cellular Component GO terms. The proteins detected in both ectosomes and exosomes-enriched fractions (total constitutive EV proteins) were grouped using GO terms related to cellular component analysis process using DAVID (Huang da et al., 2009). The graph shows the percentage of proteins identified by mass spectrometry that fall into designated GO category relative to the total number of proteins in the category. GO FAT was used to minimize the redundancy of general GO terms in the analysis. Categories with enrichment greater than 4% are shown.

In order to determine the proteomic profile of microglia-derived EVs we used a LC-MS label free approach and analyzed four independent experiments. A total of 140 and 142 proteins were detected respectively in ectosomes and exosomes produced by unstimulated microglia, of which 69 proteins were common between the two vesicle populations (∼47% overlap) (**Figure 2A**). The set of proteins uniquely identified in ectosomes or exosomes along with common proteins are shown in Supplementary Table I. Matching with proteins collected in the exosome network database ExoCarta (and its compendium Vesiclepedia) showed that a large fraction of proteins (about 88%) were already described in EVs. When the top 100 exosomal proteins were considered, almost 15% were present in EVs secreted from microglia (Supplementary Table II). Among them we found two potent anti-inflammatory mediators, AnnexinA1 and AnnexinA2, which follow an unconventional secretory pathway. Other leaderless proteins were detected in EVs derived from unstimulated microglia, such as the microglial cell type specific protein Galectin-3 (Sharma et al., 2015), Enolase, HSP90 or GAPDH (Supplementary Table I), supporting a role for EVs in unconventional protein secretion.

**FIGURE 3:**
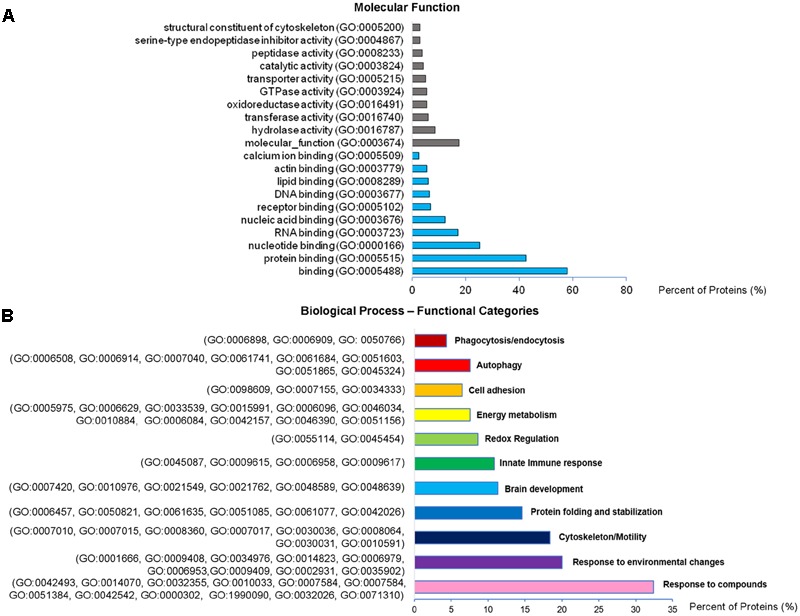
Molecular function and Biological Process analysis of constitutive EVs. **(A)** Analysis of Molecular Function GO terms of total EV proteins. **(B)** Total EV proteins were grouped using (GO) terms related to Biological process analysis process using DAVID and shown in Supplementary Table III. Biological process GO terms falling into categories with similar/related function relevant in microglia were further clustered and shown in the column charts list.

**FIGURE 4:**
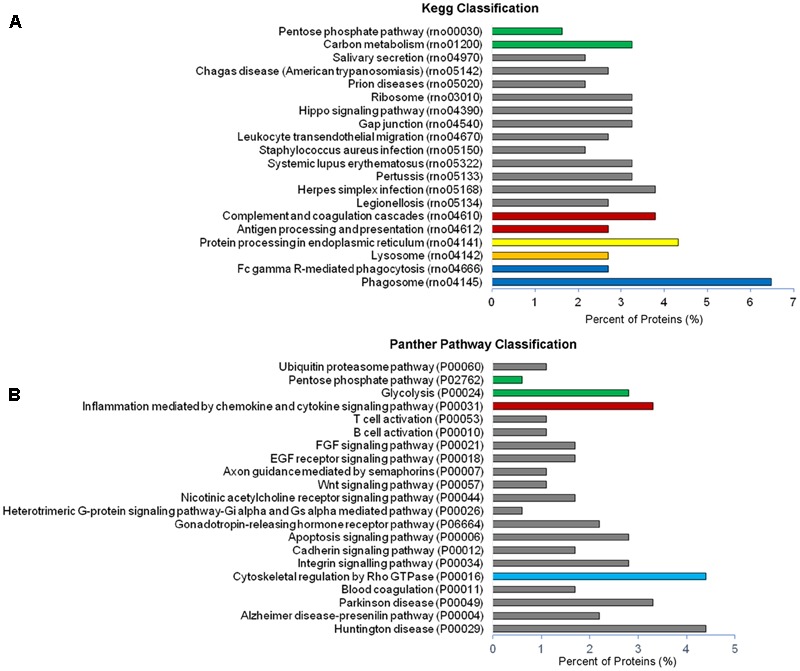
Pathway analysis of constitutive EVs. **(A)** Histogram presentation of the KEGG pathway categories of total EVs. **(B)** Histogram presentation of panther GO term analysis of pathways.

Analysis of GO terms showed that the cellular component terms “extracellular region (part),” “membrane-bounded vesicles,” “extracellular vesicles,” and “extracellular exosome” are the highest enriched fractions (above 60%) of constitutive EVs (ectosomes and exosomes in total), followed by “cytosol” and “extracellular space,” which is consistent with the vesicular and extracellular nature of EV proteins (**Figure 2B**). GO analysis of molecular functions showed that binding to protein and RNA/nucleotide are major categories, in line with adhesive properties of EVs and their content of genetic materials (**Figure 3A**). GO analysis of biological processes (Supplementary Table III) revealed terms related to response to compounds (∼32%), response to environmental changes (∼20%), cytoskeleton/motility (∼18%), protein folding and stabilization (∼15%), brain development (∼11%), innate immune response (∼11%), redox regulation (∼9%), and energy metabolism (∼8%) as predominant categories (**Figure 3B**). These functional categories reflect the surveying action of microglia and their role in brain development and homeostasis. Other important, albeit less abundant, functional categories included cell-cell adhesion (∼6%), fundamental for EV interaction with target cells, and autophagy-lysosomal pathway (∼7%) which, together with phagocytosis/endocytosis (4%), may reflect constitutive degradative activity of microglia (**Figure 3B** and Supplementary Table III). Twenty pathways were identified in constitutive EVs using the KEGG analysis. Among them “Phagosome,” “Protein processing in endoplasmic reticulum,” “Complement and coagulation cascades,” “Antigen processing and presentation,” “Lysosome,” which further highlight the degradative potential of microglial EVs as well as their role in antigen presentation and immune response (**Figure 4A**). Moreover, panther GO pathway classification revealed “Cytoskeleton regulation by Rho GTPase” and “Inflammation-mediated by chemokine and cytokine signaling pathway” among more abundant pathways, in line with immune surveillance functions of microglia (**Figure 4B** and Supplementary Table IV).

Among proteins of constitutive EVs not previously reported in ExoCarta and Vesiclepedia we found IL-18 receptor, a molecule formerly described in microglia (Prinz and Hanisch, 1999), and other microglia-enriched proteins: the degradative enzyme lysozyme C, the solute carrier family 23 (Sharma et al., 2015) and the WASL-interacting protein family member 1 (Wipf1), a protein that plays a role in the reorganization of the actin cytoskeleton (Supplementary Table V). Furthermore, we detected the centrosomal protein Cep162, the cytosolic enzyme G6PDX that participates in the pentose phosphate pathway and the reactive oxygen species-producing enzyme NADPH (nicotinamide adenine dinucleotide phosphate) oxidase 1, recently reported to mediate microglia-dependent synaptic dysfunction in experimental multiple sclerosis (Di Filippo et al., 2016), the Slingshot 3 phosphatase, a protein highly abundant in neurons but also present in microglia (Sharma et al., 2015), and the olfactory receptor Olr262, a G-coupled receptor, which is not expected to be functionally relevant in microglial cells (Supplementary Table V).

### ATP Stimulation Modifies the Proteome of Microglia-Derived EVs

**FIGURE 5:**
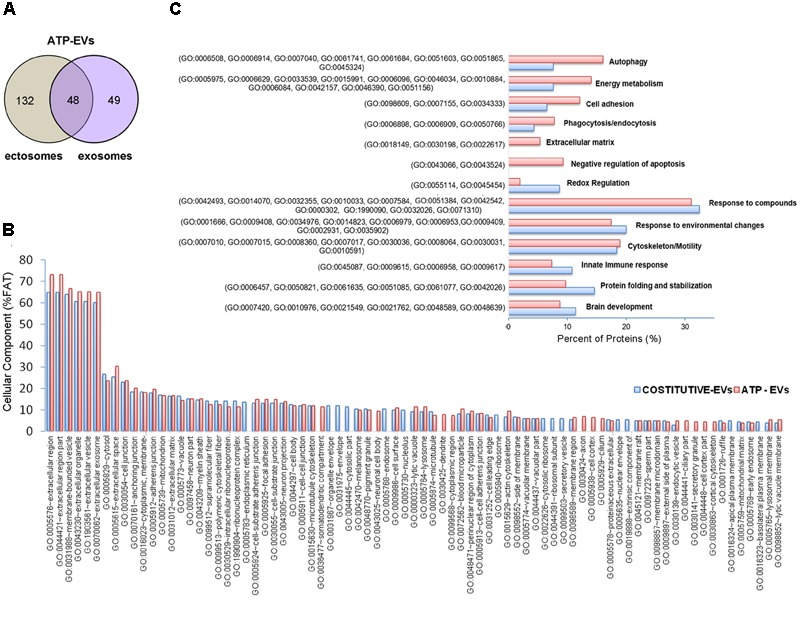
Comparative analysis of the proteome of constitutive EVs and ATP-EVs. **(A)** Venn diagram of the numerical values for common and unique proteins present in ectosomes (gray) and exosomes (violet) produced by microglia upon ATP stimulation. **(B)** Analysis of Cellular Component GO terms of ATP-EV proteins (red) relative to constitutive EV proteins (blue) as in **Figure 2B**. The graphs show the percentage of proteins identified by mass spectrometry that fall into the designated category relative to the total number of protein in the category (% FAT). GO FAT was used to minimize the redundancy of general GO terms in the analysis. Categories with enrichment greater than 4% are shown. **(C)** Histograms representation of functional categories of ATP-EV proteins (red) relevant in microglia relative to constitutive EV proteins (blue) as in **Figure 3B**.

We next analyzed the proteome of EVs secreted by microglia upon ATP stimulation (ATP-EVs). 180 and 97 proteins were found in ectosomes and exosomes respectively, of which 48 proteins were common, confirming a significant overlap (∼40%) between the two vesicle populations (**Figure 5A** and Supplementary Table VI). Proteins uniquely identified in ectosomes or in exosomes released either constitutively or under ATP stimulation, are shown in **Table 1**. These proteins may represent ectosomal and exosomal markers for microglia-derived EVs.

**Table 1:** Proteins uniquely identified in microglia-derived ectosomes or exosomes.

Markers for Microglia-derived EVs			
**Ectosome-Markers**		**Exosome-Markers**	
**Entry ID**	**Protein name**	**Entry ID**	**Protein name**

G3V6D3	ATP synthase subunit beta (Atp5b)	D3ZTH8	Uncharacterized protein (LOC689899)
G3V904	Phospholipase D family, member 4 (Pld4)	D4A6G6	Uncharacterized protein (LOC100362339)
M0R5A9	Uncharacterized protein (Dennd5b)	F1LIUW7	Myristoylated alanine-rich C-kinase substrate (Marcks)
M0RBJ7	Complement C3 (C3)	F7FEZ6/Q5I0M7	Heterogeneous nuclear ribonucleoprotein A1 (Hnrnpa2b1)
088797-2	Disabled homolog 2 (Dab2)	G3V7C6	Tubulin beta chain (Tubb4b)
P00564	Creatine kinase M-type (Ckm)	G3V7N9	Complement C1q subcomponent subunit B (C1qb)
P00787	Cathepsin B (Ctsb)	G3V8C3	Vimentin (Vim)
P04785	Protein disulfide-isomerase (P4hb)	P05370	Glucose-6-phosphate 1-dehydrogenase (G6pdx)
P10960	Prosaposin (Sulfated glycoprotein 1) (Psap)	P05982	NAD(P)H dehydrogenase 1 (Nqo1)
P11598	Protein disulfide-isomerase A3 (Pdia3)	P0CG51	Polyubiquitin-B (Ubb)
P12346	Serotransferrin (Tf)	P13471	40S ribosomal protein S14 (Rps14)
P17132/Q9JJ54	Heterogeneous nuclear ribonucleoprotein D0 (Hnrnpd)	P18588	Interferon-induced GTP-binding protein Mx1 (Mx1)
P24090	Alpha-2-HS-glycoprotein (Ahsg)	P31720	Complement C1q subcomponent subunit A (C1qa)
P62815	V-type proton ATPase subunit B, brain isoform (Atp6v1b2)	P31722	Complement C1q subcomponent subunit C (C1qc)
P68035	Actin, alpha cardiac muscle 1 (Actc1)	P45592	Cofilin-1 (Cfl1)
P70600-3	Protein-tyrosine kinase 2-beta (Ptk2b)	P62914	60S ribosomal protein L11 (Rpl11)
Q5FVQ0	Zinc transporter ZIP8 (Slc39a8)	P63039	60 kDa heat shock protein, mitochondrial (Hspd1)
Q5U1Y2	Ras-related C3 botulinum toxin substrate 2 (Rac2)	P85108	Tubulin beta-2A chain (Tubb2a)
Q5XIS1	Protein phosphatase Slingshot homolog 3 (Ssh3)	Q00715	Histone H2B type 1 (H2B1)
Q63081	Protein disulfide-isomerase A6 (Pdia6)	Q3MIE4	Synaptic vesicle membrane protein VAT-1 homolog (Vat1)
Q68FR6	Elongation factor 1-gamma (Eef1g)	Q4KLH6-2	Centrosomal protein of 162 kDa (Cep162)
Q6AXU4	E3 ubiquitin-protein ligase RNF181 (Rnf181)	Q5XI38	Lymphocyte cytosolic protein 1 (Lcp1)
Q6P0K8	Junction plakoglobin (Jup)	Q5XIN6	LETM1 and EF-hand domain-containing protein 1, mitochondrial (Letm1)
Q6P7C7	Transmembrane glycoprotein NMB (Gpnmb)	Q62667	Major vault protein (Mvp)
Q80ZA3	Alpha-2 antiplasmin (Serpinfl Dmrs91 rCG 34442)	Q63507	60S ribosomal protein L14 (Rpl14)
Q91ZN1	Coronin-1A (Coro1a)	Q6AYC4	Macrophage-capping protein (Capg)
Q9QY16-3	ATP-dependent RNA helicase DDX25 (Ddx25)	Q6AYZ1	Tubulin alpha-1C chain (Alpha-tubulin 6) (Tuba1c)
Q9R1T3	Cathepsin Z (Ctsz)	Q6URK4-2	Heterogeneous nuclear ribonucleoprotein A3 (Hnrnpa3)
		Q7TMC7	Ab2-417 (Cc1-8) (Tf)
		Q7TP54	Protein FAM65B (Fam65b)
		M0R5V7/Q6IE52	Murinoglobulin-2 (Mug2)

**FIGURE 6:**
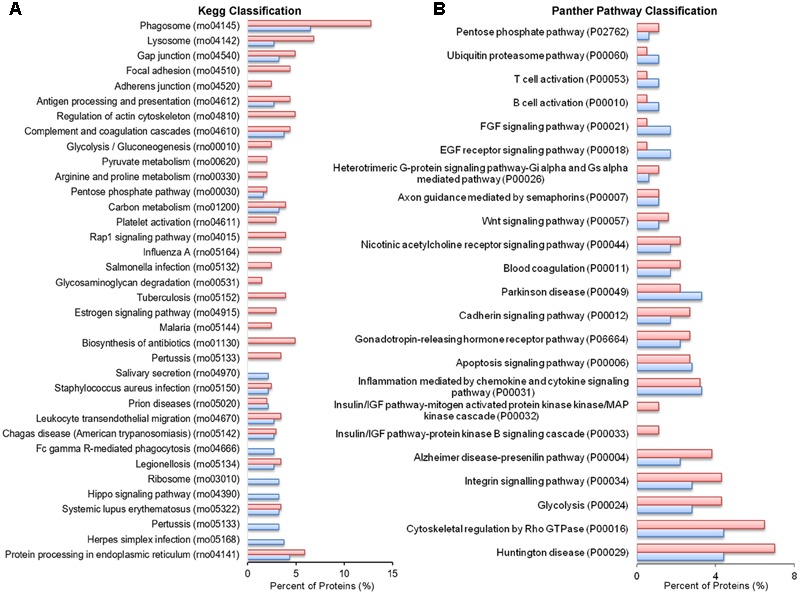
Comparative pathway analysis of constitutive EVs and ATP-EVs. **(A)** Histogram presentation of the KEGG pathways in ATP-EVs (red) versus constitutive EVs (blue). **(B)** Histogram presentation of Panther GO term analysis of pathways in ATP–EVs (red) versus constitutive EVs (blue).

No significant changes were detected in GO cellular component terms in ATP-EVs versus constitutive EVs (**Figure 5B**). However, we found substantial increases in GO biological process terms related to autophagy-lysosomal pathway (+129%), energy metabolism (+143%), cell adhesion (+100%) and phagocytosis and endocytosis (+100%), along with the appearance of new GO terms, including “Extracellular matrix” and “Apoptosis” (**Figure 5C** and Supplementary Table III). The fraction of proteins involved in redox regulation was reduced (-78%), while the fraction of proteins involved in response to compounds (∼31%), response to environmental changes (∼17%) and cytoskeleton/motility (∼19%) remained substantially unchanged (**Figure 5C** and Supplementary Table III). KEGG analysis confirmed the increase in degradative pathways (“Phagosome,” “Lysosome”) and cell adhesion pathways (“Gap-junction,” “Focal adhesion” and “Adherens junction”). It also revealed an increase in “Antigen processing and presentation” and “Regulation of actin cytoskeleton” pathways with no significant changes in “Complement and coagulation cascades” pathway (**Figure 6A**). Importantly, KEGG analysis showed differences in energy metabolism, with the appearance of specific metabolic pathways, namely “Glycolysis/Gluconeogenesis,” “Pyruvate metabolism,” and “Arginine and proline metabolism” and increased fraction of proteins involved in “Pentose phosphate pathway” and “Carbon metabolism.” Panther GO pathway classification confirmed major changes in metabolic pathways in ATP-EVs versus constitutive EVs, with a strong increase in “Glycolysis” and in “Pentose phosphate pathway” (+54% and +83% respectively) (**Figure 6B** and Supplementary Table IV), as well as in “Cytoskeletal regulation by Rho GTPase” and “Integrin signaling pathway.”

The complete list of proteins specifically associated to the ATP treatment (125 proteins), not present in constitutive EVs, are shown in Supplementary Table VII, of which 41 proteins are metabolic proteins.

### ATP-EVs Have Stronger Impact on the Activation State of Recipient Astrocytes

Higher content of proteins involved in extracellular matrix organization and cell adhesion suggested that ATP-EVs might adhere stronger to target cells compared to constitutive EVs. In addition, more abundant representation of proteins involved in antigen presentation and in cellular metabolism suggested that ATP-EVs may have a greater influence on target cells. We addressed these hypotheses using rat primary astrocytes as recipient cells, which were previously shown by us to interact with microglia-derived EVs (Prada et al., 2016) and to be activated by ATP-EVs (Verderio et al., 2012).

**FIGURE 7:**
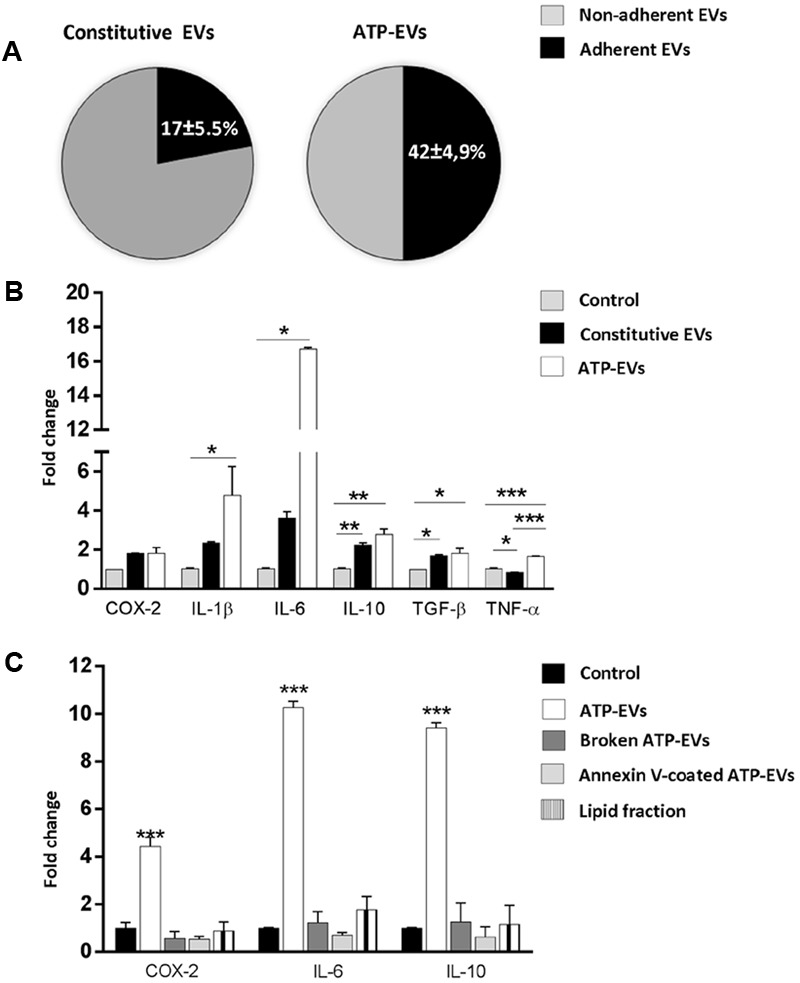
ATP-EVs activate recipient astrocytes stronger. **(A)** Pie charts show the percentage of adhesion to astrocytes of constitutive EVs and ATP-EVs produced by microglia (Constitutive EVs versus ATP-EVs, unpaired *t*-test *P* = 0.0278; *N* = 3). **(B)** q-PCR for COX-2, IL-1β, IL-6, IL-10, TNF-α and TGF-β in astrocytes exposed to constitutive EVs or ATP-EVs for 48hr (COX2: Kruskal–Wallis One-way ANOVA *P* = 0.071; IL-1β: Kruskal–Wallis One-way ANOVA *P* = 0.004 followed by Dunn’s multiple comparisons test; IL-6: Kruskal–Wallis One-way ANOVA *P* = 0.004, followed by Dunn’s multiple comparisons test; IL-10: One-way ANOVA *P* = 0.0014 followed by Tukey’s multiple comparisons test; TGF-β One-way ANOVA *P* = 0,0143 followed by Tukey’s multiple comparisons test; TNF-α: One-way ANOVA *P* = 0,0002 followed by Tukey’s multiple comparisons test; *N* = 3). **(C)** q-PCR for COX-2, IL-6, and IL-10 in control astrocytes, cells exposed for 48 h to intact ATP-EVs, broken ATP-EVs, ATP-EVs pre-incubated with annexin-V or EV lipids. (COX-2: One-way ANOVA *P* < 0.0001 Tukey’s multiple comparisons test; IL-6: One-way ANOVA *P* < 0.0001 Tukey’s multiple comparisons test; IL-10: One-way ANOVA *P* < 0.0001 Tukey’s multiple comparisons test). Data are representative of three independent experiments.

We first quantified vesicle adhesion by delivering constitutive EVs or ATP-EVs to astrocytes by optical tweezers and monitoring EV-astrocyte contact by time-lapse microscopy (three independent experiments) (Prada et al., 2016). After addition to the cultures, EVs still suspended in the medium were trapped by the IR laser tweezers and kept in contact with astrocytes for 30 s. The trapping laser was then switched off to prove EV adhesion. We found that 42 ± 4.9% of ATP-EVs adhered to astrocytes (*n* = 34), while a significant smaller percentage (17 ± 5.5%) of constitutive EVs bound to the astrocyte surface (*n* = 37) (**Figure 7A**). Next we analyzed by q-PCR the expression of few activation markers in astrocytes exposed to constitutive EVs or ATP-EVs (ectosomes) derived from equal number of donor microglia for 48 h. A stronger upregulation of IL-1β, IL-6 and TNF-α was observed in astrocytes exposed to ATP-EVs compared to constitutive EVs, supporting a role for the proteins uniquely present in ATP-EVs in the response of recipient astrocytes (**Figure 7B**). Incubation of astrocytes with the same amount of ectosomes produced either constitutively or under ATP stimulation excluded that changes in ectosome production may account for the stronger response of astrocytes to ATP-EVs (Supplementary Figure 2).

We finally explored how ATP-EVs (ectosomes) modulate astrocyte activity. First, we pretreated ATP-EVs with Annexin-V to cloak PS residues on EV membrane and prevent EV-astrocyte contact (Prada et al., 2016). Untreated ATP-EVs were used as positive control. Cloaking PS abolished IL-6 and IL-10 induction in astrocytes, revealing that EV-astrocyte contact is necessary for astrocyte activation (**Figure 7C**). Second, ATP-EVs were broken by freeze and thaw and the surface components of EVs were separated from EV luminal cargo by ultracentrifugation, as previously defined (Antonucci et al., 2012). Broken EVs did not retain the capacity to activate astrocytes (**Figure 7C**). Finally, we extracted and sonicated lipids from ATP-EVs to obtain unilamellar lipid vesicles (Antonucci et al., 2012), which were delivered to astrocytes. We found that EV lipids did not induce significant alterations of astrocyte transcripts (**Figure 7C**). Collectively these findings revealed that the luminal cargo (proteins or RNA) of ATP-EVs is responsible for the astrocyte reaction.

## Discussion

We performed a comparative analysis of the protein composition of the two main populations of microglia-derived EVs, i.e., quite large ectosomes, shed from the plasma membrane, and smaller exosomes originating from the endosomal compartment (multivesicular bodies). Ectosomes were separated from exosomes using a classical differential ultracentrifugation protocol (Bianco et al., 2009; Gabrielli et al., 2015). As a source of EVs we used primary cultured microglia maintained *in vitro* in the absence of stimulus or exposed to ATP, a well-known molecule promoting microglia activation (Domercq et al., 2013) and increasing EV production (Bianco et al., 2005b) (Bianco et al., 2009). Of note, our microglial cultures express several ATP receptors including P2Y12, a key marker that distinguish microglia from peripheral monocytes and other immune cells (Butovsky et al., 2014). To avoid cell damage, we stimulated microglia with ATP for only 1h and isolated the EVs released into the extracellular medium during this time period. Due to the shortness of the protocol and the limited expansion of primary microglia, small vesicles batches could be generated, limiting detection of low abundant proteins and quantitative analysis of EV proteome. Despite these limitations, our proteomic analysis allowed the characterization of hundreds of proteins and provided new insights on the content of microglia-derived EVs and their potential to influence the response of recipient cells. Most importantly, it offered new clues on microglia response to ATP.

### Ectosomes or Exosomes Contain Both Common and Unique Proteins

We found that exosomes and ectosomes, either constitutively released or under ATP stimulation, have a set of specific proteins but share a substantial fraction of proteins. This proteome overlap may derive, at least in part, from the isolation procedure used to collect EVs, which does not allow a precise separation of the vesicles. Accordingly, TRPS analysis showed that many ectosomes and exosomes have similar size, albeit peak sizes of the two vesicle fractions are distinct.

Among proteins present in ectosomes-enriched fraction we found 28 specific proteins of which Slingshot 3 phosphatase and ATP-dependent RNA helicase DDX25 were not described before in EVs (based on ExoCarta and Vesiclepedia). The two proteins may be used in future studies as selective markers of ectosomes released from microglia. With respect to exosome-specific proteins we found several C1q subunits.

### Balanced Expression of Inflammatory and Protective Immune Proteins in Constitutive EVs

The proteome of constitutive EVs comprises many microglia-enriched or microglia-specific proteins, reflecting the proteome (and function) of parent cells (IL-18 receptor, Galectin-3, CD14, Lysozyme C, Ferritin, Hexosaminidase Subunit Beta, Wipfi, C1q). IL-18 receptor attenuates the release of pro-inflammatory cytokines (Prinz and Hanisch, 1999), while Galectin-3 sustains microglia activation in the brain, by acting as an endogenous ligand for TLR4 (Burguillos et al., 2015). CD14 acts as co-receptor for LPS. Lysozyme C plays a role in response against bacterial infection and Ferritin has antioxidative properties. Mutations of Hexosaminidase Subunit Beta (HexB) result in the neurodegenerative gangliosidosis Sandhoff disease (Hickman et al., 2013). Wipf1 plays a role in the reorganization of the actin cytoskeleton, which is crucial for the movement of microglial processes and phagocytosis. Finally, the complement factor C1q is released from microglia during brain development (Lui et al., 2016), localizes to inappropriate synapses and acts as a tag for synaptic pruning (Stevens et al., 2007; Stephan et al., 2012), although the mechanisms allowing synapses identification remain still unclear. The presence of C1q in constitutive exosomes suggests that they may serve as vehicles to deliver the complement factors to aberrant synapses. Selective delivery of C1q may be achieved through the interaction of exosomal and synaptic adhesion proteins. Galectin-3 (via integrins), AnnexinA2, Plectin, and Tyrosine 3-monooxygenase/tryptophan 5-monooxygenase activation protein zeta (Ywhaz) are among the exosomal proteins which might promote or stabilize contact between small vesicles and synapses. Importantly, EVs may have an intrinsic capacity to move to reach their synaptic target without the need for any moving fluid or passive transport, as previously suggested (Cvjetkovic et al., 2017), as they contain multiple proteins that regulate actin filament dynamics and reorganization (Cofilin-1, Coronin 1A, Tropomodulin 2, Plectin, Wipf1, Slingshot protein phosphatase 3).

Consistently with a possible role of microglial EVs in refinement of neuronal circuits, constitutive EVs also contain proteins involved in brain development (NSF attachment protein alpha, Dihydropyrimidinase-like 2 and Myristoylated alanine rich protein kinase C substrate), regulation of neuron projection (Serpin family F member 1), as well as lysosomal enzymes (Cathepsin Z, B and Phosphoinositide-3-kinase, regulatory subunit 4), which may mediate proteolysis and degradation of aberrant synapses. Further experiments are required to test this intriguing hypothesis, which goes beyond the aim of this study.

Exosome-associated C1q (and other immune proteins) may also play a key role in microglia-astrocyte signaling. Indeed C1q is one of the three essential microglial factors recently described to be responsible for the transformation of astrocytes from trophic to reactive cells (Liddelow et al., 2017). However, our q-PCR results show that constitutive EVs cause mild upregulation of inflammatory markers and also increase the expression of the pro-regenerative markers IL-10 and TGF-β in astrocytes, suggesting that the activity of the immune proteins may be balanced by protective molecules of EVs. Among them we have identified AnnexinA1, a potent anti-inflammatory agent, that downregulates the inflammatory response in experimental models of acute (Gastardelo et al., 2009; Girol et al., 2013), chronic (Oliani et al., 2008; Dalli et al., 2010), and systemic (Damazo et al., 2005) inflammation, and AnnexinA2, which facilitates release of anti-inflammatory cytokines and has a role in host defense against infection (Zhang et al., 2015). Both Annexins were recently detected in the secretome of pro-regenerative M2 macrophages, which promote resolution of inflammation (de Torre-Minguela et al., 2016) in association with PS externalization, a process linked to EV biogenesis (Turola et al., 2012). Together with our proteomic data, these previous findings suggest that Annexins exploit EVs to be released constitutively from immune cells and influence the biological activity of EVs.

### The Proteome of EVs Reflects Microglia Response to ATP

We provide evidence that the proteome of EVs released under ATP stimulation is largely distinct from that of constitutive vesicles.

We found a strong increase in the fraction of proteins associated to autophagy-lysosomal pathway. The Lysosomal-associated membrane protein 1 (Lamp1), Cathepsin D and C, Valosin-containing protein (Vcp) and CD68, a marker of microglial activation, were exclusively present in ATP-EVs. Increased content of degradative enzymes highlights the degradation potential of EVs released from ATP-stimulated microglia. It also reflects possible enhancement of degradative pathways in microglia to meet enhanced synaptic pruning in response to ATP. Importantly, several proteins uniquely detected in ATP-EVs may indeed facilitate C1q delivery to aberrant synapses via EVs and their consequent elimination. Proteins controlling extracellular matrix organization, such as Fibulin 1 (Fbln1), Cartilage oligomeric matrix protein (Comp), Plasminogen and the Matricellular proteins thrombospondin 1 and 4 may pave the way of ATP-EVs toward synapses, while Vinculin and Fermt3, proteins essential in the organization of focal adhesions, may enhance stabilization of the contact between ATP-EVs and synapses. The capping actin protein Capzb, Cap1 and ARP2 actin related protein might contribute to changes in EV morphology and motility (Prada et al., 2016; Cvjetkovic et al., 2017).

The largest difference between the proteome of ATP-EVs and constitutive EVs was related to proteins involved in cellular metabolism. More than 60% of ATP-EV metabolic proteins were not present in constitutive vesicles and “cell metabolism” was the most abundant pathway of ATP-EV specific proteins. They included several enzymes necessary for glycolysis (Glucose-6-phosphate isomerase -Gpi-), lactate production (Lactate dehydrogenase A -Ldha-, Malate dehydrogenase 2 -Mdh2-), the oxidative branch of the pentose phosphate pathway (Tranketolase), glutamine metabolism (Glutamate dehydrogenase 1) and fatty acid synthesis (Acetyl-CoA carboxylase beta -Acacb-). Collectively these changes may reflect an increase in microglia glycolysis and fatty acid synthesis, and in glutamine metabolism, which may serve to replenish levels of TCA cycle metabolites. Upregulation of mRNAs for the glycolytic enzyme PFKFB3, for glucose transporter SLC2A1 and for fatty acid enzyme Fasn in donor cells exposed to ATP is consistent with this possibility. This metabolic change may conserve/generate adequate pool of fatty acids for enabling membrane synthesis that is necessary to enhance routine ATP-dependent microglia behavior such as process scanning and phagocytic activity (Grabert et al., 2016). Metabolic enzymes (Enolase, Glyceraldehyde 3-phosphate dehydrogenase and Pyruvate kinase) were previously reported in exosomes constitutively released from N9 murine microglial cells (Potolicchio et al., 2005). However, their abundant expression likely reflected altered metabolism of the immortalized cell line.

Previous studies demonstrated that ectosome biogenesis evoked by ATP is calcium- and P2X_7_ receptor- dependent (Bianco et al., 2005a; Pizzirani et al., 2007) and occurs from specific plasma membrane domains, the lipid rafts, where P2X_7_ receptor localizes (Bianco et al., 2009). Shedding typically involves a budding process, in which surface blebs selectively accumulate cellular constituents that are then packaged into MVs (Thomas and Salter, 2010). Following P2X7 receptor activation, cytoskeleton/membrane proteins interacting with the long cytoplasmic C-terminus of the receptor could be recruited and sorted into EVs, thus controlling the protein cargoes of ATP-EVs. Accordingly, we found that ATP-EVs contain cytoskeletal proteins and chaperones previously shown to interact with the P2X7 C-terminus (Kim et al., 2001; Gu et al., 2009). This sorting mechanism may be consistent with the proposed role of lipid rafts in setting up platforms to concentrate into MVs proteins destined to secretion (Del Conde et al., 2005).

### Stronger Impact of ATP-EVs on Receiving Astrocytes

Here we show that EVs secreted from microglia under ATP stimulation have higher impact on the activation state of recipient astrocytes, compared to constitutive EVs, and that proteins (and/or RNAs) differentially sorted into the vesicle lumen under ATP stimulation rather than surface components account for the astrocyte reaction. However, how cargoes of ATP-EVs mediate astrocyte response and whether EV internalization is necessary for astrocyte activation still remains unknown. Importantly, ATP-EVs upregulate in astrocytes both the anti-inflammatory cytokine IL-10 and the pro-inflammatory cytokine IL-6, ruling out the acquisition of a detrimental phenotype.

Augmented sorting of metabolic enzymes in ATP-EVs opens the possibility that EVs may function as independent metabolic units (Iraci et al., 2017) and have the potential to increase sugar based energy outside mitochondria in recipient cells. In astrocytes glycolysis is stimulated by increased extracellular K+ (Bittner et al., 2011), following neuronal activity, and permits release of pyruvate or lactate to support axonal function. Thus, we can speculate that ATP-EVs, through astrocyte activation, may be beneficial to neurons and favor neuronal firing. However, further studies are necessary to verify the impact of ATP-EVs on astrocyte metabolism and to define the contribution of ATP-EV proteome in the metabolic changes, which are out of the scope of this study.

The presence in ATP-EVs of proteins promoting neurite outgrowth and synaptogenesis, i.e., Trombospondin 1 and 4 (Arber and Caroni, 1995; Eroglu et al., 2009) together with proteins which negatively regulate neuron apoptosis, suggest that microglia-derived EVs may also have direct protective action toward neurons. This is in agreement with previous evidence showing that exosomes produced by other glial cells, i.e., oligodendrocytes, enhance neuronal stress tolerance and promote neuronal survival (Fruhbeis et al., 2013; Frohlich et al., 2014).

## Ethics Statement

All the experimental procedures followed the guidelines established by the European Legislation (Directive 2010/63/EU) and the Italian Legislation (L.D. no. 26/2014). It was also approved by the Italian Ministry of Health and the Bioethical Committee of the University of Milan.

## Author Contributions

FD performed LC-MS and data analysis, ML analyzed proteomic data and performed q-PCR analysis and optical tweezers experiments, IP isolated EV samples and contributed to the study design, MG performed EV quantification by qNano. PJ established mixed glial cell cultures and primary microglia cultures and helped with EV isolation. DC supervised optical tweezer experiments. JF to proteomic analysis. IF supervised proteomic analysis. JV contributed to the study design and revised the text. CV supervised the whole work and wrote the manuscript. All authors revised and approved the final version of the manuscript.

## Conflict of Interest Statement

The authors declare that the research was conducted in the absence of any commercial or financial relationships that could be construed as a potential conflict of interest.

## Footnotes

1 http://proteomecentral.proteomexchange.org

2 http://david.abcc.ncifcrf.gov

3 http://www.pantherdb.org

4 http://www.agbase.msstate.edu

5 http://www.genome.jp/kegg

## Abbreviations

ATP-EVs: EVs produced from ATP-stimulated microglia
DAVID: Database for Annotation, Visualization, and Integrated Discovery
EVs: extracellular vesicles
ExoCarta: exosome network database
FA: formic acid
FDR: False discovery rates
GO: gene ontology
IAA: 3-indole acetic acid
KEGG: Kyoto Encyclopedia of Genes and Genome
LC-MS: liquid chromatography-mass spectrometry
MVs: microvesicles
NCE: normalized collision energy
PANTHER: Protein ANalysis THrough Evolutionary Relationships
PS: phosphatidylserine
TRPS: tunable resistive pulse sensing

## References

[B1] Adinolfi E. Pizzirani C. Idzko M. Panther E. Norgauer J. Di Virgilio F. (2005). P2X(7) receptor: death or life? *Purinergic Signal.* 1 219–227. 10.1007/s11302-005-6322-x 18404507 PMC2096546

[B2] Antonucci F. Turola E. Riganti L. Caleo M. Gabrielli M. Perrotta C. (2012). Microvesicles released from microglia stimulate synaptic activity via enhanced sphingolipid metabolism. *EMBO J.* 31 1231–1240. 10.1038/emboj.2011.489 22246184 PMC3297996

[B3] Arber S. Caroni P. (1995). Thrombospondin-4, an extracellular matrix protein expressed in the developing and adult nervous system promotes neurite outgrowth. *J. Cell Biol.* 131 1083–1094. 10.1083/jcb.131.4.1083 7490284 PMC2200004

[B4] Barenholz Y. Gibbes D. Litman B. J. Goll J. Thompson T. E. Carlson R. D. (1977). A simple method for the preparation of homogeneous phospholipid vesicles. *Biochemistry* 16 2806–2810. 10.1021/bi00631a035 889789

[B5] Bianco F. Fumagalli M. Pravettoni E. D’ambrosi N. Volonte C. Matteoli M. (2005a). Pathophysiological roles of extracellular nucleotides in glial cells: differential expression of purinergic receptors in resting and activated microglia. *Brain Res. Brain Res. Rev.* 48 144–156. 10.1016/j.brainresrev.2004.12.004 15850653

[B6] Bianco F. Pravettoni E. Colombo A. Schenk U. Moller T. Matteoli M. (2005b). Astrocyte-derived ATP induces vesicle shedding and IL-1 beta release from microglia. *J. Immunol.* 174 7268–7277. 15905573 10.4049/jimmunol.174.11.7268

[B7] Bianco F. Perrotta C. Novellino L. Francolini M. Riganti L. Menna E. (2009). Acid sphingomyelinase activity triggers microparticle release from glial cells. *EMBO J.* 28 1043–1054. 10.1038/emboj.2009.45 19300439 PMC2664656

[B8] Bittner C. X. Valdebenito R. Ruminot I. Loaiza A. Larenas V. Sotelo-Hitschfeld T. (2011). Fast and reversible stimulation of astrocytic glycolysis by K+ and a delayed and persistent effect of glutamate. *J. Neurosci.* 31 4709–4713. 10.1523/JNEUROSCI.5311-10.2011 21430169 PMC6622916

[B9] Burguillos M. A. Svensson M. Schulte T. Boza-Serrano A. Garcia-Quintanilla A. Kavanagh E. (2015). Microglia-secreted galectin-3 acts as a toll-like receptor 4 ligand and contributes to microglial activation. *Cell Rep.* 10.1016/j.celrep.2015.02.012 [Epub ahead of print], 25753426

[B10] Butovsky O. Jedrychowski M. P. Moore C. S. Cialic R. Lanser A. J. Gabriely G. (2014). Identification of a unique TGF-beta-dependent molecular and functional signature in microglia. *Nat. Neurosci.* 17 131–143. 10.1038/nn.3599 24316888 PMC4066672

[B11] Buttgereit A. Lelios I. Yu X. Vrohlings M. Krakoski N. R. Gautier E. L. (2016). Sall1 is a transcriptional regulator defining microglia identity and function. *Nat. Immunol.* 17 1397–1406. 10.1038/ni.3585 27776109

[B12] Casano A. M. Albert M. Peri F. (2016). Developmental apoptosis mediates entry and positioning of microglia in the zebrafish brain. *Cell Rep.* 16 897–906. 10.1016/j.celrep.2016.06.033 27425604

[B13] Casano A. M. Peri F. (2015). Microglia: multitasking specialists of the brain. *Dev. Cell* 32 469–477. 10.1016/j.devcel.2015.01.018 25710533

[B14] Cocucci E. Meldolesi J. (2015). Ectosomes and exosomes: shedding the confusion between extracellular vesicles. *Trends Cell Biol.* 25 364–372. 10.1016/j.tcb.2015.01.004 25683921

[B15] Corriden R. Insel P. A. (2012). New insights regarding the regulation of chemotaxis by nucleotides, adenosine, and their receptors. *Purinergic Signal.* 8 587–598. 10.1007/s11302-012-9311-x 22528684 PMC3360098

[B16] Cvjetkovic S. J. Jeremic V. L. Tiosavljevic D. V. (2017). Knowledge and attitudes toward vaccination: a survey of Serbian students. *J. Infect. Public Health* 10 649–656. 10.1016/j.jiph.2017.05.008 28669785

[B17] Dalli J. Rosignoli G. Hayhoe R. P. Edelman A. Perretti M. (2010). CFTR inhibition provokes an inflammatory response associated with an imbalance of the annexin A1 pathway. *Am. J. Pathol.* 177 176–186. 10.2353/ajpath.2010.091149 20489160 PMC2893661

[B18] Damazo A. S. Yona S. D’acquisto F. Flower R. J. Oliani S. M. Perretti M. (2005). Critical protective role for annexin 1 gene expression in the endotoxemic murine microcirculation. *Am. J. Pathol.* 166 1607–1617. 10.1016/S0002-9440(10)62471-6 15920146 PMC1602430

[B19] Davalos D. Grutzendler J. Yang G. Kim J. V. Zuo Y. Jung S. (2005). ATP mediates rapid microglial response to local brain injury in vivo. *Nat. Neurosci.* 8 752–758. 10.1038/nn1472 15895084

[B20] de Torre-Minguela C. Barbera-Cremades M. Gomez A. I. Martin-Sanchez F. Pelegrin P. (2016). Macrophage activation and polarization modify P2X7 receptor secretome influencing the inflammatory process. *Sci. Rep.* 6:22586. 10.1038/srep22586 26935289 PMC4776275

[B21] Del Conde I. Shrimpton C. N. Thiagarajan P. Lopez J. A. (2005). Tissue-factor-bearing microvesicles arise from lipid rafts and fuse with activated platelets to initiate coagulation. *Blood* 106 1604–1611. 10.1182/blood-2004-03-1095 15741221

[B22] Di Filippo M. De Iure A. Giampa C. Chiasserini D. Tozzi A. Orvietani P. L. (2016). Persistent activation of microglia and NADPH oxidase [corrected] drive hippocampal dysfunction in experimental multiple sclerosis. *Sci. Rep.* 6:20926. 10.1038/srep20926 26887636 PMC4757867

[B23] Di Virgilio F. (2007). Purinergic signalling in the immune system. A brief update. *Purinergic Signal.* 3 1–3. 10.1007/s11302-006-9048-5 18404413 PMC2096765

[B24] Domercq M. Vazquez-Villoldo N. Matute C. (2013). Neurotransmitter signaling in the pathophysiology of microglia. *Front. Cell Neurosci.* 7:49 10.3389/fncel.2013.00049 PMC3630369 23626522

[B25] Dou Y. Wu H. J. Li H. Q. Qin S. Wang Y. E. Li J. (2012). Microglial migration mediated by ATP-induced ATP release from lysosomes. *Cell Res.* 22 1022–1033. 10.1038/cr.2012.10 22231629 PMC3367529

[B26] Eroglu C. Allen N. J. Susman M. W. O’rourke N. A. Park C. Y. Ozkan E. (2009). Gabapentin receptor alpha2delta-1 is a neuronal thrombospondin receptor responsible for excitatory CNS synaptogenesis. *Cell* 139 380–392. 10.1016/j.cell.2009.09.025 19818485 PMC2791798

[B27] Farber K. Kettenmann H. (2006). Purinergic signaling and microglia. *Pflugers. Arch.* 452 615–621. 10.1007/s11302-006-9048-5 16791619

[B28] Frohlich D. Kuo W. P. Fruhbeis C. Sun J. J. Zehendner C. M. Luhmann H. J. (2014). Multifaceted effects of oligodendroglial exosomes on neurons: impact on neuronal firing rate, signal transduction and gene regulation. *Philos. Trans. R. Soc. Lond. B Biol. Sci.* 369:20130510. 10.1098/rstb.2013.0510 25135971 PMC4142031

[B29] Fruhbeis C. Frohlich D. Kuo W. P. Amphornrat J. Thilemann S. Saab A. S. (2013). Neurotransmitter-triggered transfer of exosomes mediates oligodendrocyte-neuron communication. *PLOS Biol.* 11:e1001604. 10.1371/journal.pbio.1001604 23874151 PMC3706306

[B30] Gabrielli M. Battista N. Riganti L. Prada I. Antonucci F. Cantone L. (2015). Active endocannabinoids are secreted on extracellular membrane vesicles. *EMBO Rep.* 16 213–220. 10.15252/embr.201439668 25568329 PMC4328748

[B31] Gastardelo T. S. Damazo A. S. Dalli J. Flower R. J. Perretti M. Oliani S. M. (2009). Functional and ultrastructural analysis of annexin A1 and its receptor in extravasating neutrophils during acute inflammation. *Am. J. Pathol.* 174 177–183. 10.2353/ajpath.2009.080342 19095957 PMC2631330

[B32] Ginhoux F. Greter M. Leboeuf M. Nandi S. See P. Gokhan S. (2010). Fate mapping analysis reveals that adult microglia derive from primitive macrophages. *Science* 330 841–845. 10.1126/science.1194637 20966214 PMC3719181

[B33] Girol A. P. Mimura K. K. Drewes C. C. Bolonheis S. M. Solito E. Farsky S. H. (2013). Anti-inflammatory mechanisms of the annexin A1 protein and its mimetic peptide Ac2-26 in models of ocular inflammation in vivo and in vitro. *J. Immunol.* 190 5689–5701. 10.4049/jimmunol.1202030 23645879

[B34] Grabert K. Michoel T. Karavolos M. H. Clohisey S. Baillie J. K. Stevens M. P. (2016). Microglial brain region-dependent diversity and selective regional sensitivities to aging. *Nat. Neurosci.* 19 504–516. 10.1038/nn.4222 26780511 PMC4768346

[B35] Gu B. J. Rathsam C. Stokes L. Mcgeachie A. B. Wiley J. S. (2009). Extracellular ATP dissociates nonmuscle myosin from P2X(7) complex: this dissociation regulates P2X(7) pore formation. *Am. J. Physiol. Cell Physiol.* 297 C430–C439. 10.1152/ajpcell.00079.2009 19494237

[B36] Hickman S. E. Kingery N. D. Ohsumi T. K. Borowsky M. L. Wang L. C. Means T. K. (2013). The microglial sensome revealed by direct RNA sequencing. *Nat. Neurosci.* 16 1896–1905. 10.1038/nn.3554 24162652 PMC3840123

[B37] Honda S. Sasaki Y. Ohsawa K. Imai Y. Nakamura Y. Inoue K. (2001). Extracellular ATP or ADP induce chemotaxis of cultured microglia through Gi/o-coupled P2Y receptors. *J. Neurosci.* 21 1975–1982. 11245682 10.1523/JNEUROSCI.21-06-01975.2001 PMC6762617

[B38] Hooper C. Sainz-Fuertes R. Lynham S. Hye A. Killick R. Warley A. (2012). Wnt3a induces exosome secretion from primary cultured rat microglia. *BMC Neurosci.* 13:144. 10.1186/1471-2202-13-144 23173708 PMC3541220

[B39] Huang da W. Sherman B. T. Lempicki R. A. (2009). Systematic and integrative analysis of large gene lists using DAVID bioinformatics resources. *Nat. Protoc.* 4 44–57. 10.1038/nprot.2008.211 19131956

[B40] Iraci N. Gaude E. Leonardi T. Costa A. S. H. Cossetti C. Peruzzotti-Jametti L. (2017). Extracellular vesicles are independent metabolic units with asparaginase activity. *Nat. Chem. Biol.* 13 951–955. 10.1038/nchembio.2422 28671681 PMC5563455

[B41] Kim M. Jiang L. H. Wilson H. L. North R. A. Surprenant A. (2001). Proteomic and functional evidence for a P2X7 receptor signalling complex. *EMBO J.* 20 6347–6358. 10.1093/emboj/20.22.6347 11707406 PMC125721

[B42] Liddelow S. A. Guttenplan K. A. Clarke L. E. Bennett F. C. Bohlen C. J. Schirmer L. (2017). Neurotoxic reactive astrocytes are induced by activated microglia. *Nature* 541 481–487. 10.1038/nature21029 28099414 PMC5404890

[B43] Lui H. Zhang J. Makinson S. R. Cahill M. K. Kelley K. W. Huang H. Y. (2016). Progranulin deficiency promotes circuit-specific synaptic pruning by microglia via complement activation. *Cell* 165 921–935. 10.1016/j.cell.2016.04.001 27114033 PMC4860138

[B44] Matcovitch-Natan O. Winter D. R. Giladi A. Vargas Aguilar S. Spinrad A. Sarrazin S. (2016). Microglia development follows a stepwise program to regulate brain homeostasis. *Science* 353:aad8670. 10.1126/science.aad8670 27338705

[B45] Nimmerjahn A. Kirchhoff F. Helmchen F. (2005). Resting microglial cells are highly dynamic surveillants of brain parenchyma in vivo. *Science* 308 1314–1318. 10.1126/science.1110647 15831717

[B46] Ohsawa K. Irino Y. Sanagi T. Nakamura Y. Suzuki E. Inoue K. (2010). P2Y12 receptor-mediated integrin-beta1 activation regulates microglial process extension induced by ATP. *Glia* 58 790–801. 10.1002/glia.20963 20091784

[B47] Oliani S. M. Ciocca G. A. Pimentel T. A. Damazo A. S. Gibbs L. Perretti M. (2008). Fluctuation of annexin-A1 positive mast cells in chronic granulomatous inflammation. *Inflamm. Res.* 57 450–456. 10.1007/s00011-008-7222-7 18827967

[B48] Paolicelli R. C. Bolasco G. Pagani F. Maggi L. Scianni M. Panzanelli P. (2011). Synaptic pruning by microglia is necessary for normal brain development. *Science* 333 1456–1458. 10.1126/science.1202529 21778362

[B49] Pizzirani C. Ferrari D. Chiozzi P. Adinolfi E. Sandona D. Savaglio E. (2007). Stimulation of P2 receptors causes release of IL-1beta-loaded microvesicles from human dendritic cells. *Blood* 109 3856–3864. 10.1182/blood-2005-06-031377 17192399

[B50] Pocock J. M. Kettenmann H. (2007). Neurotransmitter receptors on microglia. *Trends Neurosci.* 30 527–535. 10.1016/j.tins.2007.07.007 17904651

[B51] Potolicchio I. Chitta S. Xu X. Fonseca D. Crisi G. Horejsi V. (2005). Conformational variation of surface class II MHC proteins during myeloid dendritic cell differentiation accompanies structural changes in lysosomal MIIC. *J. Immunol.* 175 4935–4947. 10.4049/jimmunol.175.8.4935 16210595

[B52] Prada I. Amin L. Furlan R. Legname G. Verderio C. Cojoc D. (2016). A new approach to follow a single extracellular vesicle-cell interaction using optical tweezers. *Biotechniques* 60 35–41. 10.2144/000114371 26757810

[B53] Prada I. Furlan R. Matteoli M. Verderio C. (2013). Classical and unconventional pathways of vesicular release in microglia. *Glia* 61 1003–1017. 10.1002/glia.22497 23625857

[B54] Preissler J. Grosche A. Lede V. Le Duc D. Krugel K. Matyash V. (2015). Altered microglial phagocytosis in GPR34-deficient mice. *Glia* 63 206–215. 10.1002/glia.22744 25142016

[B55] Prinz M. Hanisch U. K. (1999). Murine microglial cells produce and respond to interleukin-18. *J. Neurochem.* 72 2215–2218. 10.1046/j.1471-4159.1999.0722215.x 10217305

[B56] Sharma K. Schmitt S. Bergner C. G. Tyanova S. Kannaiyan N. Manrique-Hoyos N. (2015). Cell type- and brain region-resolved mouse brain proteome. *Nat. Neurosci.* 18 1819–1831. 10.1038/nn.4160 26523646 PMC7116867

[B57] Sieger D. Moritz C. Ziegenhals T. Prykhozhij S. Peri F. (2012). Long-range Ca2+ waves transmit brain-damage signals to microglia. *Dev. Cell* 22 1138–1148. 10.1016/j.devcel.2012.04.012 22632801

[B58] Stephan A. H. Barres B. A. Stevens B. (2012). The complement system: an unexpected role in synaptic pruning during development and disease. *Annu. Rev. Neurosci.* 35 369–389. 10.1146/annurev-neuro-061010-113810 22715882

[B59] Stevens B. Allen N. J. Vazquez L. E. Howell G. R. Christopherson K. S. Nouri N. (2007). The classical complement cascade mediates CNS synapse elimination. *Cell* 131 1164–1178. 10.1016/j.cell.2007.10.036 18083105

[B60] Thomas L. M. Salter R. D. (2010). Activation of macrophages by P2X7-induced microvesicles from myeloid cells is mediated by phospholipids and is partially dependent on TLR4. *J. Immunol.* 185 3740–3749. 10.4049/jimmunol.1001231 20709956 PMC2933301

[B61] Turola E. Furlan R. Bianco F. Matteoli M. Verderio C. (2012). Microglial microvesicle secretion and intercellular signaling. *Front. Physiol.* 3:149. 10.3389/fphys.2012.00149 22661954 PMC3357554

[B62] Verderio C. Muzio L. Turola E. Bergami A. Novellino L. Ruffini F. (2012). Myeloid microvesicles are a marker and therapeutic target for neuroinflammation. *Ann. Neurol.* 72 610–624. 10.1002/ana.23627 23109155

[B63] Zhang S. Yu M. Guo Q. Li R. Li G. Tan S. (2015). Annexin A2 binds to endosomes and negatively regulates TLR4-triggered inflammatory responses via the TRAM-TRIF pathway. *Sci. Rep.* 5:15859. 10.1038/srep15859 26527544 PMC4630631

