# Supplementary material for: ATP Modifies the Proteome of Extracellular Vesicles Released by Microglia and Influences Their Action on Astrocytes
Source: Front Pharmacol. 2017 Dec 13;8:910. doi: 10.3389/fphar.2017.00910 (PMC5733563; doi:10.3389/fphar.2017.00910)
Supplement: Supplementary file 2 [file Image_1.PDF]

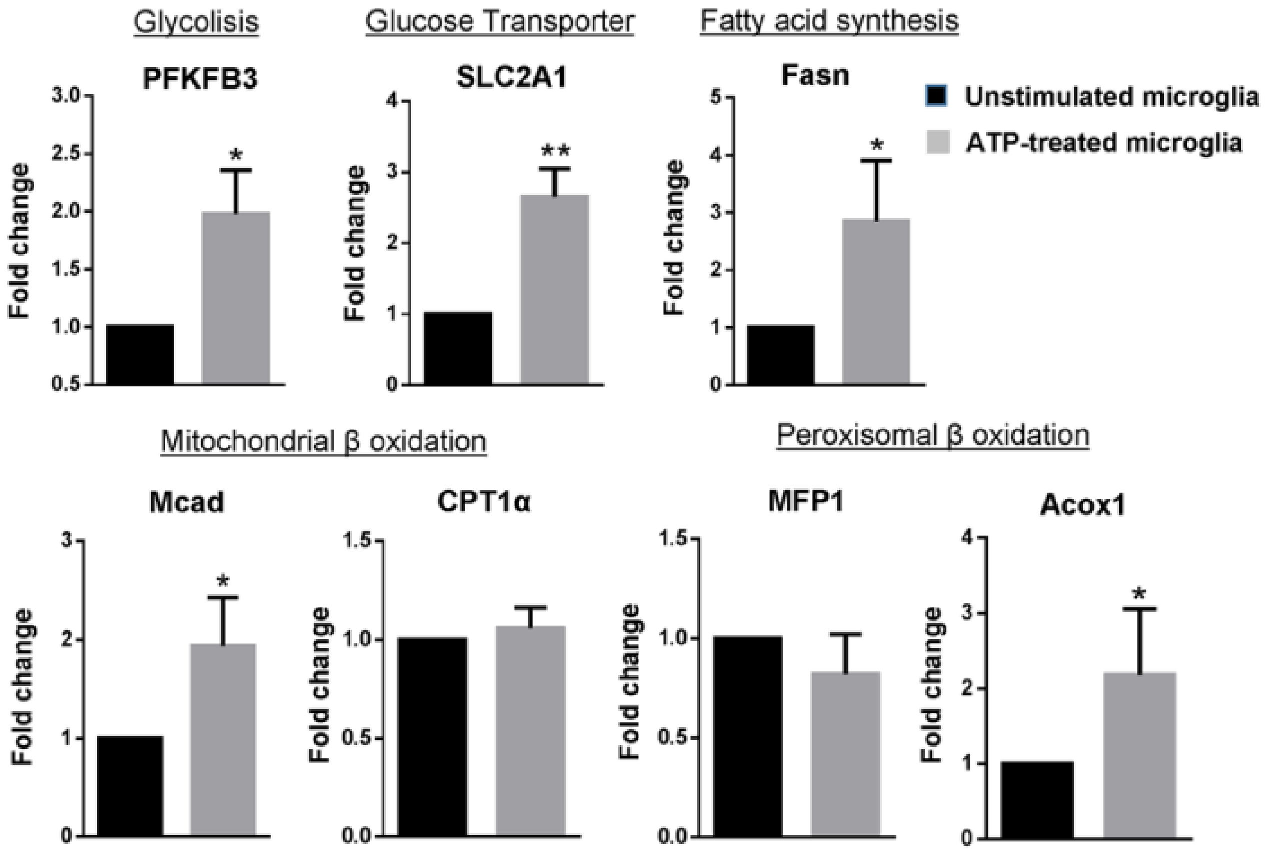

Supplementary Figure 1. Expression of metabolic genes in unstimulated and ATP-treated microglia. Q-PCR analysis for the glycolytic marker PFKFB3 (6-phosphofructo-2-kinase/fructose-2,6-biphosphatase 3), the glucose transporter 1 SLC2A1, the fatty acid synthetic enzyme Fasn, the mitochondrial β-oxidation markers Mcad (Medium-chain acyl-CoA dehydrogenase) and CPT1α (Carnitine palmitoyltransferase I), and the peroxisomal β-oxidation markers MFP1 (Multifunctional protein 1) and Acox1 (Peroxisomal acyl-coenzyme A oxidase 1) (PFKFB3: Unpaired t test P=0,0421; SLC2A1: Unpaired t test P=0,0059; Fasn: Mann Whitney test P=0,0286 ; Mcad: Mann Whitney test P=0,0286; CPT1α: Mann Whitney test P=0,6825; MFP1: Mann Whitney test P=0,6825; Acox1: Mann Whitney test P=0,0286; N=4).
