# Supplementary material for: ATP Modifies the Proteome of Extracellular Vesicles Released by Microglia and Influences Their Action on Astrocytes
Source: Front Pharmacol. 2017 Dec 13;8:910. doi: 10.3389/fphar.2017.00910 (PMC5733563; doi:10.3389/fphar.2017.00910)
Supplement: Supplementary file 3 [file Image_2.PDF]

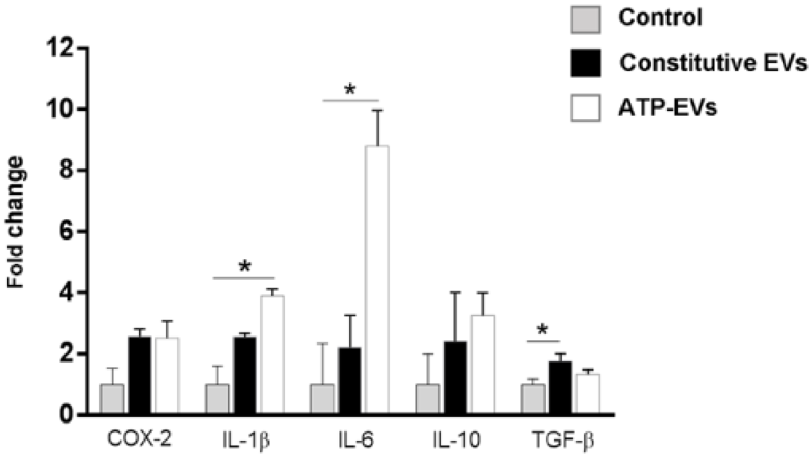

Supplementary Figure 2. Expression of activation markers in astrocytes exposed to Constitutive EVs or ATP-EVs. Q-PCR analysis for COX-2, IL-1β, IL-6, IL-10, and TGF-β in astrocytes exposed to the same amount of ectosomes ($1X10^8$ particles/ml) released from unstimulated microglia or ATP-treated microglia (COX-2: Kruskal-Wallis One Way ANOVA P=0,0464; IL-1□: Kruskal-Wallis One Way ANOVA P=0,0250 followed by Dunn's multiple comparisons test; IL-6: Kruskal-Wallis One Way ANOVA P=0,0036 followed by Dunn's multiple comparisons test; IL-10: Kruskal-Wallis One Way ANOVA P=0,2643; TGF-β: Kruskal-Wallis One Way ANOVA P=0,0071 followed by Dunn's multiple comparisons test).
